# Supplementary material for: Maximizing multi-reaction dependencies provides more accurate and precise predictions of intracellular fluxes than the principle of parsimony
Source: PLoS Comput Biol. 2023 Sep 18;19(9):e1011489. doi: 10.1371/journal.pcbi.1011489 (PMC10538754; doi:10.1371/journal.pcbi.1011489)
Supplement: Attachment — Submitted filename: Reponse Letter.docx [file pcbi.1011489.s019.docx]

**Reviewer #1:**

The challenge of estimating accurate metabolic flux maps is addressed in this MS (PCOMPBIOL-D-22-01807) with a new constraint-based method. Accurate flux maps are important for industrial biotechnology, especially for engineered strains or those adaptively evolved in the laboratory to become specialist at the desired production tasks. Genetically, these are strains that have mutations under positive selection pressure with strongly causal mutations. This characteristic i[s] in contrast to WT strains that normally have mutations under negative selection pressure resulting more generalist phenotypes, and thus are probably more flexible use of their metabolic networks so that accurate flux maps are not as critical to know. Genome-scale models can be used to compute optimal growth and production rates based on flux-balance analysis (FBA) using measured uptake rates. FBA calculations do not give a unique solution for the metabolic flux maps, calling for the imposition of additional constraints to confine possible flux maps. pFBA was developed as a proxy for most parsimonious uses of the metabolic proteome by minimizing the length of the flux vector. Albeit ‘fewer’ and more constrained, the optimal pFBA solutions are not unique. pFBA has become popular method as it is easy to implement and seen to generate physiologically meaningful solutions, consistent with its underlying principles.

The authors aim to generate an improvement of and an alternative to pFBA. They use comparisons to experimental fluxomic data from laboratory-evolved strains as a standard for comparison.

**Response: Thanks for the brief summary of the main contribution. While we agree with the claim that positive selection leads usually to more constrained flux space, this information does not enter in the model generation or in the comparison of the two approach – pFBA and cbFBA. We note that both approaches use the same input – the model together with growth constraints, leading to a fair comparison.**

It needs to be explicitly recognized that there are experimental errors in these flux estimates. Furthermore, it needs to be recognized that most of the fluxes, reported as fluxomics, are computed from relatively few experimental measurements, typically using a reduced stoichiometric model. Although, I believe that the data the authors use in this paper are from studies that used comprehensive metabolic networks to computational estimate the fluxomic state of the cells.

**Response: We would like to stress that all of our analyses either used the lower and upper bounds from the confidence intervals, or did a comparison of the means from the established confidence intervals from the constraint-based vs. MFA estimations. That said, the used experimental flux distributions from MFA were derived using a large-scale model, which is the reason why we selected them in the analyses. We are aware that model size used for fitting of labelling patterns in MFA can drastically affect the flux estimates (e.g. see the work of (Gopalakrishnan & Maranas, 2015)). Therefore, our analyses are already in line with the suggestion from the reviewer.**

Nevertheless, unlike sequence-based datatypes, metabolomics and fluxomes are datatypes ‘etherical’ in nature as they are very hard to estimate accurately in vivo, and modelers should be aware of these limitations.

**Response: We are fully aware of the issues and challenges associated with flux estimation from labelling data, and we have included an additional sentence to this effect in the introduction of the updated version of the manuscript.**

The core premise of the proposed and developed new methods is to use topological features of the metabolic network through a decomposition of the stoichiometric matrix, as S=YA, to compute flux states that require minimal regulation, driven by ‘multi-reaction dependences (unfortunately this concept is not clearly and explicity described to be accessible to a general reader).’ The reader is introduced to the method with:

“We postulate that upstream transcriptional and transcriptional regulation of real-world metabolic networks leads to the maximization of multi-reaction dependencies as a principle of metabolic functionality; these multi-reaction dependencies would decrease the need for fine-tuned regulation of reaction fluxes.”

**Response: In the updated version of the manuscript, we detail the concept of balanced complexes based on the decomposition of the stoichiometric matrix as S = YA. This we in turn use to better illustrate and specify the notion of multi-reaction dependencies.**

Based on this postulate, the authors formulate the complex-balanced FBA (cbFBA) method, which takes a while to adsorb and understand. I strongly recommend that the authors try to make the concept more accessible and perhaps use a nice graphic/visual to explain it. This visual should ideally include the principles of pFBA as a reference and show how the new methods generates deviation from this state that is understandable based on the underlying principle that they base the method on. Without such clear explanation, the readership of the article and user of the new method may be very few.

**Response: We have modified Fig. 1 that now also provides a comparison of the proposed cbFBA with pFBA. We have used the example to also illustrate the concept of balanced complexes and the implications thereof.**

The Lewis lab at UCSD maintains a collection of FBA methods: https://smex-ctp.trendmicro.com:443/wis/clicktime/v1/query?url=http%3a%2f%2fcobramethods.wikidot.com%2f&umid=1f747d4f-b048-474f-9b37-1b419d2b075e&auth=682db514602deaff239ec316aa24e93dba6f746c-e87472e2860cb29ef96c220bb75eb3d077153208. The cbFBA method, if published, will likely become a part of this collection, and COBRA computational toolboxes. This collection was first published in NRM in, I think 2012. The cbFBA method should be placed and categorized within this structure of methods so the prospective users know how it fits in and understand what its pros and cons are.

**Response: We would like to thank the reviewer for this constructive suggestion. We have perused the collection of FBA methods and have placed cbFBA in its context. As a result, we have provided a reference to the study in NRM (Lewis et al., 2012)** **in the introduction.**

Fig 1 is not that easy to understand, and neither are the results of the computations. A small number of modelers will probably make their way through this material, but less expert reader may not. The S=YA is an interesting and thought-provoking decomposition of the stoichiometric matrix. Are the cbFBA solutions truly distinct from the space of allowable pFBA solutions? Are they unique?

**Response: We are pleased that the reviewer finds the concepts behind cbFBA thought provoking. We show that the space of allowable solutions differs between pFBA and cbFBA because their comparison based on FVA shows clear differences. Moreover, we already showed in Table S1 that the solutions from cbFBA, while more constrained than those from pFBA, are still not unique (again from the differences in the lower and upper bounds from FVA for cbFBA). Nevertheless, we streamlined the text to provided additional details and clarify the similarities and differences between pFBA and cbFBA.**

pFBA is based on a simple and easy to understand principle, minimization of flux-load, with computations that require minimum number of experimentally	measured variables and are a simple confinement of unbounded FBA solutions. The comparison of the pFBA and cbFBA solution spaces should be compared. If they overlap, then the methods have something in common. If they are fully disjointed, then their different characteristics should be delineated. Figure 2 is uninspiring and does not do this subject matter justice.
This methods once clearly described, characterized, and distinguished from pFBA represents publishable material and is suitable for PLoS Comp Bio.

**Response: Following the reviewer’s suggestion, we added additional analyses of the existing results by delineating reactions / pathways between pFBA and cbFBA that show overlapping vs. distinct flux ranges. In addition, we have provided an additional figure, Fig. 3, in the updated version of the manuscript, to better illustrate the differences and similarities across reactions in different pathways.**

**Reviewer #2: Summary**:

The author proposed a new variation of flux balance analysis by decomposing the stoichiometric matrix and using one of the decomposed matrices to maximize multi-reaction dependencies. Although the work is not a major improvement from the pFBA algorithm, still it looks interesting. There are a few points that need to be clarified. Besides, the authors can use a smaller model (maybe E. coli core model) to clearly demonstrate a case where cbFBA outsmarts pFBA. Overall, the following points can be addressed to improve the manuscript.

**Response: We would like to thank the reviewer for the brief summary of the work, which is distinct in comparison to pFBA. We do not follow the suggestion to use the smaller *E. coli* core model, because we do not have flux estimates from MFA based on this model for diverse conditions. It is for this reason that we used the strains generated by ALE and their fluxes estimates from the same model used in our pFBA and cbFBA comparison. We would like to note that the yeast model is precisely smaller, as requested by the reviewer.**

**Major Critiques:**

1. While authors have made significant effort to justify the claims of the proposed cbFBA’s accuracy and precision over that of pFBA, the explicit contributions and novelty of this scheme need to be further articulated by clearly highlighting knowledge improvement useful to the scientific research community, including a soft biological analysis with relevant background knowledge, otherwise, it would be confounding our understanding of cellular metabolism.

**Response: The contribution of the work is to show that the flux distributions predicted by cbFBA are more precise and more accurate than the currently used solutions based on pFBA. This is the main achievement of this study and we further stress it in the updated version of the manuscript—particularly, the naming of the subsections of the Results.**

My strong critique would be that minimizing the conservation of multi-reaction dependencies across different flux solutions, other than the parsimony principle, may lead to biologically unrealistic or non-intuitive solutions. The parsimony principle is a widely accepted and commonly used approach in metabolic modeling, particularly with enzyme conservation – as projected application, which assumes that the cell prefers to use the minimum number of reactions or enzymes to carry out a given function. This approach is biologically plausible and has been shown to produce physiologically relevant solutions.

**Response: We do not question the parsimony principle. Our main claim, as stated in the response to the comment above, is that we identify a principle that leads to improved accuracy and precision of flux predictions over pFBA. To demonstrate these findings, we used the flux profiles from MFA together with the confidence intervals (CIs) to provide an unbiased comparison of pFBA and cbFBA. Therefore, the usage of flux estimates together with models form which they were obtained in the comparative analyses validates that imposing the principle of maximizing reaction dependencies is biologically plausible. Another reason for plausibility is that the maximization of dependencies implies modular network structure, which is prevalent for metabolic and other biological network (Barabási & Oltvai, Nat Rev Genet, 2004). We have expanded on this concept where we introduce the formulation of cbFBA, thus putting the imposed principles in perspective.**

However, minimizing the conservation of multi-reaction dependencies across various flux solutions is a less established method. It may be mathematically elegant, but it is unclear if it is biologically pertinent or consistent with our current understanding of metabolic regulation. In addition, it may generate solutions that are challenging to interpret or experimentally validate. Mismatch in flux overlaps between cbFBA and the other groups, pFBA and experimental data (lines 198–205) suggests a missing link clearly – membrane dysfunctions? Nature would not attempt to balance all complexes, except perhaps signaling molecules. Think of phosphorylation by kinases and several other factors, such as energy efficacy, resource availability, environmental conditions, etc., may also play a role. In certain pathways, maintaining a balance of metabolites and complexes may be essential, but imbalances are often necessary for proper regulation and function. In glycolysis, for instance, ATP production is favored over NADH production, resulting in an imbalanced production of these two metabolites. This imbalance is crucial for the overall function of the pathway and is controlled by numerous enzymes and feedback mechanisms. Also, this is hinged on the model’s completeness.

**Response: We find that this comment from the reviewer is likely based on misinterpretation of the concept of balanced complexes. Prompted by a comment from reviewer #1, we rewrote the section describing the concepts on which cbFBA relies, and also modified Fig. 1 to clearly illustrate the differences between pFBA and cbFBA and specifies the meaning of balanced complexes.**

Moreover, with the postulate that “upstream transcriptional and transcriptional regulation of real-world metabolic networks leads to the maximization of multi-reaction dependencies as a principle of metabolic functionality” (lines 134-136), a potential criticism of the postulate that “maximizing multi-reaction dependencies leads to decreased need for fine-tuned regulation of reaction fluxes” (lines 136-137) is that it may not account for the fact that some reactions in a metabolic network may have more important biological functions than others. For example, some reactions may be critical for cell survival or have regulatory roles, and it may be necessary to fine-tune the fluxes of these reactions even if they are not part of a multi-reaction dependency. Additionally, while linear programming can efficiently determine balanced complexes, it may not be able to capture the full complexity of metabolic networks, which can have non-linear and dynamic behaviors. Therefore, it may be important to validate the postulate with additional modeling and experimental studies. Also, the supporting validation work in Yeast showed some inconsistent trends. Authors would want to justify and should consider that supposed experimental flux estimates from the smaller network as used in this study do not necessarily reflect nature. The reverse would be the case where nature often garners more complexities than model networks.

**Response: First, we would like to stress that all constraint-based approaches are linear in nature, hence, the mentioned non-linearities will not be captured by any of this approaches (e.g. pFBA or the proposed cbFBA, as well as FBA on its own, all have linear programming formulations). Second, the very concept of multi-reaction dependencies indicates that their presence does not eliminate the need for regulation, but rather that by regulating one of the reactions in the set would indirectly regulate the fluxes of the other – a biologically plausible hypothesis in line with the mentioned modular organization in our response to the comment above. Third, flux estimates based on MFA and integration of labelling patterns are known to result in discrepant findings when different models are used – which explains the possible variation observed in the results for yeast.**

Therefore, I would recommend that the authors carefully justify and evaluate the use of this approach in their study and provide a clear comparison of the results obtained with the parsimony principle. They should also address the potential limitations and challenges of using this approach and discuss the implications of their findings for our understanding of cellular metabolism.

**Response: We have added an additional figure, Fig. 3 in the updated version of the manuscript, to provide additional analyses of reactions that show overlapping vs. distinct intervals from pFBA and cbFBA. We hope that these additional results, summarized on lines 273 – 295, provide sufficient biological insights justifying the plausibility of the proposed approach.**

2. Introduction, lines (90-91): The sentence “since the application of pFBA usually results in a substantial narrowing of the solution space as indicated by a variant of flux variability analysis” contradicts the authors' statement, in lines 30-33 of the abstract - “showed that the steady-state flux distributions resulting from cbFBA in comparison to pFBA show better agreement with experimentally measured fluxes from Escherichia coli strains and are more precise, due to the smaller space of alternative solutions”. Authors should clarify this for the benefit of readers. Moreover, lines 213-216 of result section, says otherwise. All these should be reconciled.

**Response: We do not see the contradiction, since the statement about pFBA in the introduction is in comparison to FBA, while the statement in the abstract is based on the comparison between pFBA and cbFBA. Therefore, we did not include any changes to the manuscript to this point.**

3. Methods, lines 457-469: Potential for bias. While flux sampling in this manner may be redundant, I assume the authors included it to demonstrate the accuracy of cbFBA. However, authors must consider the flux distribution of the sampling vector of random fluxes. It is assumed that authors have used normal sampling, this choice is dependent on the means of flux ranges employed and could be a redundant case, as means of flux samples would likely fall within the means of flux variability range. Consideration of an alternative case of uniform sampling, perhaps to ensure a decent coverage of the entire feasible space of solutions for a large number of samples, is also outside the scope of this paper. In addition, uniform sampling may not be optimal for extremely nonlinear or non-convex optimization problems, as it may overlook crucial regions of the feasible space. In addition to addressing the convergence issue, the popular "coordinate-hit-and-run" algorithm also addresses this one.

**Response: As mentioned above, neither of the formulations of pFBA or cbFBA are non-linear or non-convex. Hence, the comment does not correspond to what is done in the present work. Second, flux sampling is not redundant with respect to FVA, since the former provides the shape of the flux distribution in a given flux ranges, while the latter only gives insights in the flux ranges (without showing whether or not fluxes are uniformly distributed in the range). Therefore, we do not see an issue with the presented findings.**

4. Methods and Results - Statistical tests and outliers, lines 224-236 & 271-279: To clearly justify the claim that cbFBA improves accuracy, authors must provide additional information or data, bearing in mind that both the Pearson and Spearman's tests used are sensitive to outliers, which can considerably affect the strength and direction of the correlation. The possibility of skewed pFBA predictions may also be related to the biased sampling method.

**Response: All of our analyses are based on log-transformed flux values, which eliminate the issue of outliers. Nevertheless, we have updated the main text to clearly indicate the transformations used.**

5. Results and discussions, lines 333-342: The MOMA agreements are dependent on the potential bias of flux mean values. A more accurate analysis of agreement would involve the overlap of flux ranges for both methods and experiments.

**Response: We have included an analysis in which we compare flux distributions sampled from the CIs of the WT and mutants from the MFA as well as pFBA / cbFBA results and calculated the respective Euclidean distance. We performed 1000 repetitions of this procedure to obtain robust estimates of the mean and standard deviations for the Euclidean distance. Our findings support the claim in the first version of the manuscript, that the flux distributions from cbFBA are better representative for the MOMA hypothesis, widely used for simulation of mutants.**

6. Results and Discussion, Page 7, Lines 123-127: The decomposition of the S matrix into Y and A is confusing. Please provide a supplementary file and detail each step of the decomposition with an illustrated example. Besides, further discussion is needed regarding the geometrical meaning of each decomposed matrix. For instance, the singular value decomposition of a matrix can be interpreted as rotation, scaling, or rotation. A similar notion is expected for the S=YA decomposition as well.

7. Results and Discussion, Page 7, Lines 129-132: A workflow figure for the S=YA can be useful to understand the concept.

**Response: We have provided details of how these matrices are obtained in the updated manuscript, where we introduce the concepts behind cbFBA. We have provided such a figure as an illustration.**

8. Results and Discussion, Page 7, Lines 137-142: As pFBA uses the principles of minimizing the total enzyme requirements in a given metabolic network, the biology behind cbFBA is not clear. Please include your comments on that.

**Response: See our response to point 1 above.**

9. Results and Discussion, Page 10, Lines 185-190: If a reaction was blocked in the model and had experimental MFA data available, how did the authors reconcile that?

**Response: This is not possible, since MFA used with the model that has a blocked reaction will always estimate a flux of zero for that reaction.**

10. Results and Discussion, Page 10, Lines 192-194: If flux sampling analysis is performed, then what is the relevance of flux variability analysis to determine flux ranges? Please provide an explanation.

**Response: We have provided an explanation in the answer to comment 3, above.**

11. Results and Discussion, Page 10, Lines 200-209: The authors mentioned several pathways relevant to cbFBA and pFBA. However, what these pathways mean in terms of the biology of E. coli is missing. Please provide an adequate explanation of these pathways.

**Response: We have expanded briefly on the relevance of the identified pathway differences between pFBA and cbFBA for *E. coli* physiology.**

12. Results and Discussion, Page 10, Lines 217-220: It seems flux range overlap is the main selling point of the cbFBA. However, it is still not convincing the need for flux ranges, when sampling data is available. This raises the question of the better efficacy of cbFBA over pFBA. Please add discussion on this matter.

**Response: We refer the reviewer to our answer to point 3, above for the need of both approaches. We also note that the sampling approach allows us to provide a robust estimate of flux means, which is not otherwise possible from FVA alone.**

13. Materials and Methods, Page 16, Lines 412-428: Is the flux space equivalent in both the optimization problem? Please comment on that, if possible, provide proof using mathematical induction/contradiction.

**Response: No, the flux space is not the same, as illustrated by the findings from FVA.**

14. Materials and Methods, overall: Provide a detailed procedure of S=YA decomposition.
**Response: We have included this in the updated version of the manuscript and it is also available as function in our GitHub.**

**Minor Critiques:**

1. Results and Discussion, 7, Lines 115-117: the figure of the metabolic network can be improved significantly. At first look, this does not even look like a metabolic network, but rather a series of disjoint reactions. An improved version of the figure is expected.

**Response: We have streamlined and updated all figures to improve the presentation of our findings.**

2. Results and Discussion, Page 7, Line 123: “There are ten complexes” or eleven?

**Response: We thank the reviewer for spotting this typo. We have modified the example fully.**

3. Materials and Methods, overall: Please comment about the simulation platform.
**Response: We detailed the environments used for the analyses**.

4. Figure 2, Line 210 and line 227: flux concordance average Pearson correlation should be reconciled. 0.81 on figure and 0.82 in text.

**Response: We have modified, as indicated.**

**References**

Barabási, A. L., & Oltvai, Z. N. (2004). Network biology: understanding the cell’s functional organization. *Nature Reviews. Genetics*, *5*(2), 101–113. https://doi.org/10.1038/NRG1272

Gopalakrishnan, S., & Maranas, C. D. (2015). 13C metabolic flux analysis at a genome-scale. *Metabolic Engineering*, *32*, 12–22. https://doi.org/10.1016/J.YMBEN.2015.08.006

Lewis, N. E., Nagarajan, H., & Palsson, B. O. (2012). Constraining the metabolic genotype-phenotype relationship using a phylogeny of in silico methods. *Nature Reviews. Microbiology*, *10*(4), 291–305. https://doi.org/10.1038/NRMICRO2737
