# Supplementary material for: Maximizing multi-reaction dependencies provides more accurate and precise predictions of intracellular fluxes than the principle of parsimony
Source: PLoS Comput Biol. 2023 Sep 18;19(9):e1011489. doi: 10.1371/journal.pcbi.1011489 (PMC10538754; doi:10.1371/journal.pcbi.1011489)
Supplement: Attachment — Submitted filename: response_letter_2nd_revision.docx [file pcbi.1011489.s020.docx]

6. Results and Discussion, Page 7, Lines 123-127: The decomposition of the S matrix into Y
and A is confusing. Please provide a supplementary file and detail each step of the
decomposition with an illustrated example. Besides, further discussion is needed regarding
the geometrical meaning of each decomposed matrix. For instance, the singular value
decomposition of a matrix can be interpreted as

**Response**: The reviewer must have used the first rather than the revised version of the manuscript, since the lines 123-127 do not correspond to the matrix Y. In addition, the comment was not completely pasted on the system, as you can see above. Nevertheless, we decided, for full transparency, to provide the pseudocode in the methods. In addition, Figure 1, already contain a worked out example. Lastly, as noted in the first revision of the manuscript, the decomposition S = YA has to the with graph-theoretic representation of the biochemical reactions, thus fully addressing the comment from the reviewer.
